# Case report: Alternative approach for management of refractory volume overload in heart failure: usefulness of venous leg compression

**DOI:** 10.3389/fcvm.2023.1230980

**Published:** 2023-09-29

**Authors:** Carlos Collado Macián, David Pujol Pocull, Fernando Dominguez, Juan Carlos López-Azor, Pablo Garcia-Pavia, Julio Nuñez, Marta Cobo Marcos

**Affiliations:** ^1^ Department of Cardiology, Hospital Universitario Puerta de Hierro Majadahonda, (IDIPHISA), Madrid, Spain; ^2^ Centro de Investigación Biomédica en Red en Enfermedades Cardiovasculares (CIBERCV), Madrid, Spain; ^3^ Centro Nacional de Investigaciones Cardiovasculares (CNIC), Madrid, Spain; ^4^ Department of Cardiology, Hospital Clínico Universitario de Valencia, INCLIVA, Universidad de Valencia, Valencia, Spain

**Keywords:** heart failure and transplantation diuretic resistance, edema, heart failure, venous leg compression, lower limb compression bandaging

## Abstract

**Background:**

Management of patients with refractory congestion, is one of the most important challenges in the field of heart failure (HF). Diuretic therapy remains the most widely used therapy to achieve euvolemia. However, some patients experience fluid overload despite the use of high-dose diuretics and new strategies to overcome diuretic resistance are needed.

**Case Summary:**

We report an 85 years-old male patient admitted for decompensated HF with persistent tissue fluid overload (peripheral edema) for more than two weeks despite high dose of intravenous furosemide with the combination of other diuretics. At this point, we performed leg venous compression using elastic bandages for three days. After 72 h, edema disappeared, and additional weight loss was achieved (1 kg/day). No side effects were observed and the patient was discharged home euvolemic.

**Conclusion:**

Venous leg compression may be an alternative therapy in patients with persistent tissue fluid overload resistant to diuretics.

## Introduction

Fluid overload is frequent in patients with HF. A sustained increase in intravascular hydrostatic pressure and vascular permeability make fluid overload shift towards the interstitium, leading to extravascular fluid overload (1, 2). Most diuretics reduce intravascular congestion; however, hypotension and worsening renal function may limit the efficacy of diuretic therapy (3), so new strategies to overcome diuretic resistance are needed.

## Case report

We present the case of an 85-year-old male who attended our emergency department with shortness of breath. The patient had a medical history of hypertension, dyslipidemia, obesity, chronic kidney disease, chronic obstructive pulmonary disease, ischemic heart disease, and atrial fibrillation. He presented his first episode of HF with preserved ejection fraction (HFpEF) two years earlier. At that moment, screening of amyloidosis was performed, and no secondary etiologies of HFpEF were found.

His treatment included amiloride/hydrochlorothiazide 5/50 mg/24 h, furosemide 120 mg/24 h, and dapagliflozin 10 mg/24 h.

On physical examination, he presented jugular venous distension, bilateral pulmonary crackles and bilateral edema above the knees. He was hemodynamically stable, showing a blood pressure of 140/60 mmHg, a heart rate of 71 beats per minute, and an oxygen saturation of 93% without oxygen therapy. At the moment of admission, his weight was 112 kg.

Blood tests showed elevated NT-proBNP (926 pg/ml) and worsening kidney function: creatinine 2.15 mg/dl (estimated glomerular filtration rate: 27.1 ml/min/1.73 m^2^, previous value 45.5 ml/min/1.73 m^2^). Chest x-ray revealed mild bilateral pleural effusion.

Echocardiography confirmed mild global hypertrophy of the left ventricle, preserved ejection fraction, diastolic dysfunction grade II, severe dilated left atrium, without pulmonary hypertension. The inferior vena cava was enlarged (21 mm), and lung B-lines were found in 6/8 fields.

Electrocardiogram revealed atrial fibrillation at 90 beats per minute and right bundle branch block.

The patient was admitted with the diagnosis of decompensated HF, and diuretic treatment was initiated following the 2021 ESC Heart Failure Guidelines (4).

## Therapeutic intervention

An intravenous bolus of 120 mg furosemide (same dose taken orally at home) was administered.

The initial diuretic response was partially adequate (diuretic rate, 133 ml/h; urinary sodium, 56 mEq/L). However, congestion signs persisted, and the goal of 3,000 ml of diuresis per day was not achieved. Therefore, the furosemide dose was increased to 200 mg/12 h, and a sequential nephron blockade with chlorthalidone (50 mg/24 h) and dapagliflozin was implemented.

**Figure 1:**
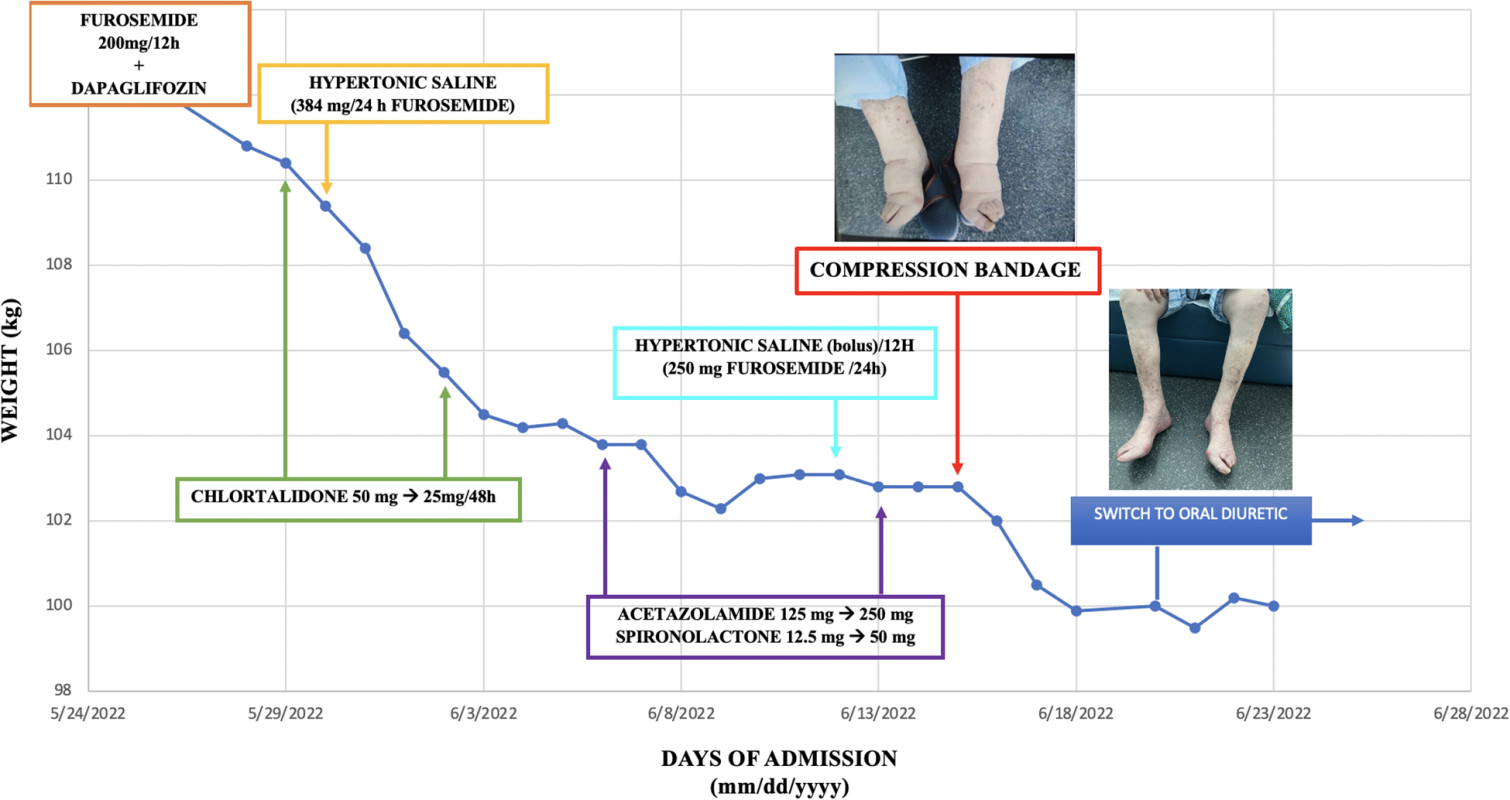
Timeline evolution of patient weight depending on the treatment administered. This timeline shows the different diuretics used during the patient's admission and their influence on weight loss. On day 20, the edema in the lower limbs was still evident; therefore, venous leg compression therapy was started for 72 h. When the bandages were removed, euvolemia was achieved, which allowed the change to oral diuretics and the patient's discharge.

Despite this diuretic scalation, diuretic response didn't improve (Figure 1), so we decided to administer a combination of intravenous furosemide with hypertonic saline solution (Table 1) to improve tissue congestion. Subsequently, the patient presented a progressive increase in daily diuresis rate (>3,000 ccs every 24 h), an improvement in kidney function (creatinine 1.8 mg/dl), and a weight loss of 1–2 kg per day (8 kg on day 11).

**Table 1:** Diuretics and their dose used in the admission.

Diuretic	Dose	Days of admission
Furosemide	Bolus (IV)^a^: 200 mg/12 h	1st→4th
	Hypertonic saline^a^ (IV): 384 mg/24 h	4th→17th
	Bolus with hypertonic saline^b^ (IV): 125 mg/12 h	17th→24th
	160 mg/24 h (PO)	25th→discharge
Chlortalidone	50 mg/24 h (PO)	3rd→6th
	25 mg/48 h (PO)	7th→discharge
Dapagliflozin Acetazolamide	10 mg/24 h (PO)	1st→discharge
	125 mg/24 h (PO)	11th→18th and 22th→discharge
	250 mg/24 h (PO)	18th→22th
Spironolactone	12.5 mg/24 h (PO)	11th→18th and 22th→discharge
	25 mg/24 h (PO)	18th→22th

The table shows the diuretics we have been used and their dose during the admission. IV: intravenous. PO: per oral.

^a^ Perfusion with hypertonic saline contains 500 mg of furosemide in 250 ml of saline solution (0.9% of sodium chloride) with two vials of NaCl 20%.

^b^ Bolus with hypertonic saline contains 125 mg of furosemide in 100 ml of saline solution (0.9% of sodium chloride) with two vials of NaCl 20%.

Despite this, the patient persisted with edema up to the knee; therefore, two new classes of diuretics (acetazolamide and spironolactone) were added, without a significant improvement in diuretic response. On day 20, the patient persisted with signs of tissue congestion and signs of intravascular depletion (IVC diameter from 20 mm on admission to 14 mm, which collapses >50%).

Therefore, we decided to apply crepe bandages to the lower limbs from the feet to the knees. The initial bandage was removed after 24 h, and a new one was placed.

After 72 h of combined diuretic and leg venous compression (LVC), the patient lost 3 kg of weight, the edema resolved, and the kidney function improved. A slight increase in NT-proBNP and in the IVC diameter (from 14 to 17 mm after 24 h) was observed as an indirect sign of intravascular repletion. After achieving euvolemia, we could switch to oral therapy and discharge the patient. After six months of follow-up in our HF multidisciplinary program, the patient is asymptomatic, with no further decompensations.

## Discussion

In this case, LVC was a helpful strategy for overcoming diuretic resistance in a patient with predominant tissue fluid overload and absence of intravascular congestion.

Multiparametric evaluation of congestion to quantify and phenotype congestion may lead to a more effective tailored approach.

Compression therapy in patients with HF has traditionally been considered a contraindication due to the hypothesis that increased cardiac preload may lead to worsening pulmonary congestion (5–7).

These hemodynamic changes have been analyzed in different studies. Derreppe et al. observed a significant increase in right atrial and pulmonary pressure after venous leg compression. Thirty minutes after withdrawing compression therapy, these parameters returned to baseline values (8).

In a study conducted by Wilputte et al., an increase in right blood pressure was observed with a transient deterioration in ventricular function secondary to an increase in preload and afterload in patients with NYHA III-IV HF (9).

Interestingly, a recent study evaluated the effect of 72 h-venous LVC according to IVC diameter in 20 subjects with ambulatory congestive HF (10). When basal IVC was <21 mm, a significant increase in the diameter of the IVC was observed in the group treated with LVC (approximately 2 mm), and this intravascular filling was associated with greater decongestion (increased natriuresis, weight loss, and peripheral edema). These results were not observed in the group with an initial vena cava diameter >21 mm.

Likewise, we observed a 3-mm increase in the diameter of the IVC after starting LVC. This was accompanied by an increase in diuresis rate and a slight increase in NT-proBNP levels.

## Conclusion

Refractory congestion, particularly when tissue fluid overload predominates, is a complex scenario in which diuretic treatment may not be able to achieve euvolemia. In our case, LVC, together with the use of diuretics, allowed the patient to reach a state of euvolemia. This therapy could have an increasing role in the management of patients with evidence of extravascular/tissue fluid overload but without intravascular overload.

## Data Availability

The original contributions presented in the study are included in the article/Supplementary Material, further inquiries can be directed to the corresponding author.
